# Life‐history traits and physiological limits of the alpine fly *Drosophila nigrosparsa* (Diptera: Drosophilidae): A comparative study

**DOI:** 10.1002/ece3.3810

**Published:** 2018-01-17

**Authors:** Martin‐Carl Kinzner, Patrick Krapf, Martina Nindl, Carina Heussler, Stephanie Eisenkölbl, Ary A. Hoffmann, Julia Seeber, Wolfgang Arthofer, Birgit C. Schlick‐Steiner, Florian M. Steiner

**Affiliations:** ^1^ Institute of Ecology University of Innsbruck Innsbruck Austria; ^2^ School of BioSciences Bio21 Institute University of Melbourne Parkville Vic. Australia; ^3^ Institute for Alpine Environment Eurac Research Bozen/Bolzano Italy

**Keywords:** Alpine species, *Drosophila*, laboratory experiments, life‐history traits, physiological limits

## Abstract

Interspecific variation in life‐history traits and physiological limits can be linked to the environmental conditions species experience, including climatic conditions. As alpine environments are particularly vulnerable under climate change, we focus on the montane‐alpine fly *Drosophila nigrosparsa*. Here, we characterized some of its life‐history traits and physiological limits and compared these with those of other drosophilids, namely *Drosophila hydei*,* Drosophila melanogaster*, and *Drosophila obscura*. We assayed oviposition rate, longevity, productivity, development time, larval competitiveness, starvation resistance, and heat and cold tolerance. Compared with the other species assayed, *D. nigrosparsa* is less fecund, relatively long‐living, starvation susceptible, cold adapted, and surprisingly well heat adapted. These life‐history characteristics provide insights into invertebrate adaptations to alpine conditions which may evolve under ongoing climate change.

## 1 INTRODUCTION

Life‐history theory forms a basic framework for interpreting reproduction, development, and lifespan of an organism (Nylin & Gotthard, 1998). Additionally, physiological limits, such as starvation resistance and heat tolerance, provide information on a species’ ecology and evolutionary adaptations (Hoffmann & Sgrò, 2011; Huey et al., 2012). While it is common to provide a molecular baseline of a new study species, for instance, through characterizing biomarkers or reference genes, it is less common to publish the baseline of its life‐history traits and physiological limits, although the latter are important for understanding species’ evolution and ecology (Hoffmann & Sgrò, 2011; Huey et al., 2012; LoPresti, Karban, Robinson, Grof‐Tisza, & Wetzel, 2016; Markow, 2015). Variation of environmental conditions might induce alternative states of life‐history traits and physiological characteristics (Karl, Stoks, De Block, Janowitz, & Fischer, 2011). For example, with increasing temperature, fecundity and body size could decrease, while development could be accelerated (Angilletta & Dunham, 2003; Kingsolver & Huey, 2008). Thus, life‐history and physiological characteristics vary within and among species depending on genetic variation and environmental factors (Hoffmann & Sgrò, 2011; Roff, 2002). Changing environments in particular cause strong selection pressures leading to potential rapid adaptation of these characteristics (Hoffmann & Sgrò, 2011; Matzkin, Watts, & Markow, 2009).

At higher altitudes, species are adapted to the harsh environmental conditions including short growing seasons, resource shortage, and extreme low and high temperatures (Franz, 1979; Hodkinson, 2005). Due to low average temperatures, oviposition and growth rate are low, the latter also resulting in long development times (Mani, 1962; Schnebel & Grossfield, 1986). Alpine species are expected to be relatively cold and heat tolerant due to the temperature extremes they encounter both during the day and throughout the year (Gaston & Chown, 1999). To our knowledge, the influence of environmental factors on life‐history strategies of alpine insects has rarely been investigated.

Recent climate change is affecting organisms, changing their behavior, physiology, and distribution (Chown et al., 2010; Hoffmann & Sgrò, 2011; IPCC, 2007). Temperature is among the most impacted environmental factors (IPCC, 2007), playing a key role in an organisms’ physiology, ecology, and evolution (Angilletta, 2009). In the Alps, climate warming has already caused physiological stress, changes in species’ diversity, and shifts in species’ ranges to higher elevation (Chen, Hill, Ohlemüller, Roy, & Thomas, 2011; Gottfried et al., 2012; Hoffmann & Sgrò, 2011; Medlock et al., 2013; Rosbakh, Bernhardt‐Römermann, & Poschlod, 2014; Svobodová et al., 2014). Ongoing climate change compels rapid adaptation of organisms (Breshears, López‐Hoffman, & Graumlich, 2011), which could occur on a phenotypic and/or genetic level if possible at all (Lande, 2009).

**Figure 1:**
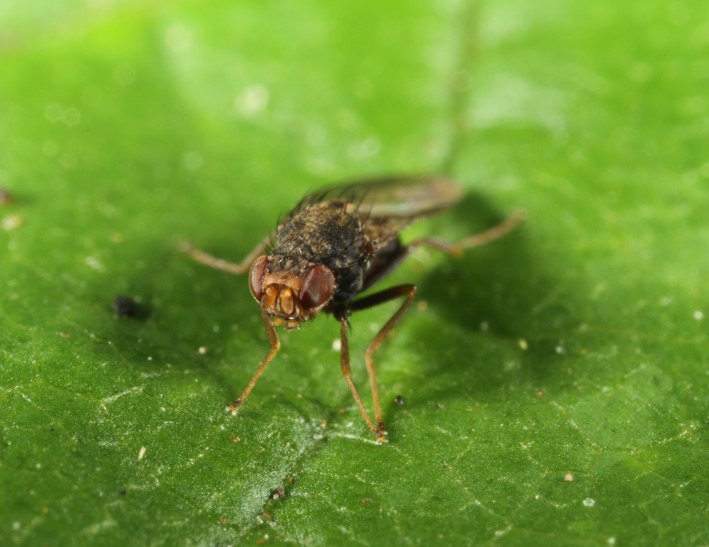
The alpine fly *Drosophila nigrosparsa*

The genus *Drosophila* contains some of the best studied animal species, whereby *Drosophila melanogaster* is in major focus of research (Hoffmann, Sørensen, & Loeschcke, 2003). So far, climate change research on Drosophilidae has rarely focused on thermal evolution of alpine species, although mountain ecosystems are predicted to be particularly vulnerable to changing temperatures (IPCC, 2007). For this reason, we are currently establishing *Drosophila nigrosparsa* (Figure 1) as an alpine study system (Austrian Science Fund, project number P 23949). *Drosophila nigrosparsa* is native to montane‐alpine regions, occurring around the timber line at about 2,000 m above sea level (a.s.l.) in Central Europe (Bächli & Burla, 1985). It uses fungal fruiting bodies as natural oviposition substrate (Kinzner et al., 2016); a specific food source of adults is not known. The exact position of this species in the *Drosophila* phylogeny is not clear, but it seems that *D. nigrosparsa* is related to the Hawaiian species (Cicconardi, Marcatili, Arthofer, Schlick‐Steiner, & Steiner, 2017). In contrast to *Drosophila alpina* (Bächli & Burla, 1985), *D. nigrosparsa* is culturable in the laboratory without an obligatory diapause, making it an ideal alpine study organism for laboratory experiments.

The main aim of this study was to test whether selected life‐history traits and physiological limits of the novel alpine model organism *Drosophila nigrosparsa* reflect the alpine environment. Thus, we characterized multiple traits of *D. nigrosparsa* under laboratory conditions, namely oviposition rate, productivity, development time, longevity, larval competition performance, starvation resistance, and heat and cold resistance. We focus on thermal limits because of increasing temperatures due to climate change. It was not our aim to examine the adaptability to climate change but to provide a baseline for future investigations. To benchmark *D. nigrosparsa*'s performance, additional *Drosophila* species with different ecological niches were tested in a subset of assays. Our goal was to directly compare our focal species with species both well characterized in former studies and common as study organisms in ecological and evolutionary experiments. *Drosophila obscura*, a species with wide temperate distribution, served as a link between the specialist, alpine‐montane *D. nigrosparsa*, the cosmopolitan *D. melanogaster*, and the tropical *Drosophila hydei* (Gibert, Moreteau, Pétavy, Karan, & David, 2001).

## 2 MATERIALS AND METHODS

### 2.1 Origin and maintenance of flies

Fly strains are named according to the first letter of the species name, and the year when the assays were carried out. All species were identified following Bächli and Burla (1985) and were kept at a 16L:8D photoperiod and ca. 70% relative humidity; rearing temperatures varied as described below. No specific permissions were required because none of the species used in this study is endangered or protected.

In 2007, *Drosophila melanogaster* (M_2015_) was captured in Melbourne (Victoria, Australia) at 20 m a.s.l. (7.82°S, 144.94°E). In 2010, *D. nigrosparsa* (N_2015_) and *D. obscura* (O_2015_) were captured at Kaserstattalm at 2,000 m a.s.l. (Tyrol, Austria, 47.13°N, 11.30°E), and *D. hydei* (H_2015_) was captured in Innsbruck at 600 m a.s.l. (Tyrol, Austria, 47.27°N, 11.35°E). A strain of each species was kept in environmental test chambers (MIR‐254, Panasonic, Etten Leur, Netherlands) at 19°C. For the larval competition experiment (see below), *D*. *subobscura* (co‐occurring with *D. nigrosparsa*) was captured at Kaserstattalm and reared as mentioned above.

In a large‐scale field survey in 2012, *Drosophila nigrosparsa* (N_2013_) was captured again at Kaserstattalm (K) but also at Pfitscherjoch (P; South Tyrol, Italy, 46.98°N, 11.68°E, 2,000 m a.s.l.). From each of the populations, 100 males and 100 females were used (N_2013_K and N_2013_P) for mass breeding. Fly stocks were kept in environmental test chambers (MLR‐352H‐PE, Panasonic, Etten Leur, Netherlands) with a diurnal temperature variation following summer measurements at 2,000 m a.s.l. in Tyrol (Austria) (M. Tratter Kinzner, M.‐C. Kinzner, R. Kaufmann, A. A. Hoffmann, W. Arthofer, B. C. Schlick‐Steiner, F. M. Steiner, unpubl.; Table S1) to provide laboratory temperature conditions as near as possible to natural conditions. The 5th generation of N_2013_K and N_2013_P was randomly split into eight replicate lines each.

To adapt flies to laboratory conditions and to check for parasites and disease (Ashburner & Roote, 2007), at least four generations were bred before flies were used in experiments. Strain M_2015_ was cultivated for 8 years before experimental use, N_2015_, O_2015_, and H_2015_ for 5 years, and N_2013_ for 1 year.

*Drosophila nigrosparsa* was reared in sterile 36‐ml glass vials on 8 ml malt medium (8.4% malt, 4.2% cornmeal, 1.3% dried yeast, 0.8% agar, 0.3% propionic acid, and 0.2% methyl‐4‐hydroxybenzoate in deionized water; modified from Lakovaara, 1969). The quality of each malt‐medium batch was tested by transferring 20 eggs into each of four randomly chosen vials. After 3–4 days of incubation, hatching success and the number of larvae alive were examined. Only media batches without dead larvae were further used. Eighty eggs or vital larvae of *D. nigrosparsa* were transferred to each vial. *Drosophila hydei*,* D. melanogaster*, and *D. obscura* were reared on a cornmeal medium (10.4% sucrose, 4.9% cornmeal, 2.6% yeast, 0.8% agar, 0.2% methyl‐4‐hydroxybenzoate, and 0.3% propionic acid in deionized water; modified from Hoffmann & Turelli, 1988). Eighty eggs or vital larvae were transferred to sterile 36‐ml glass vials containing 8 ml medium.

**Figure 2:**
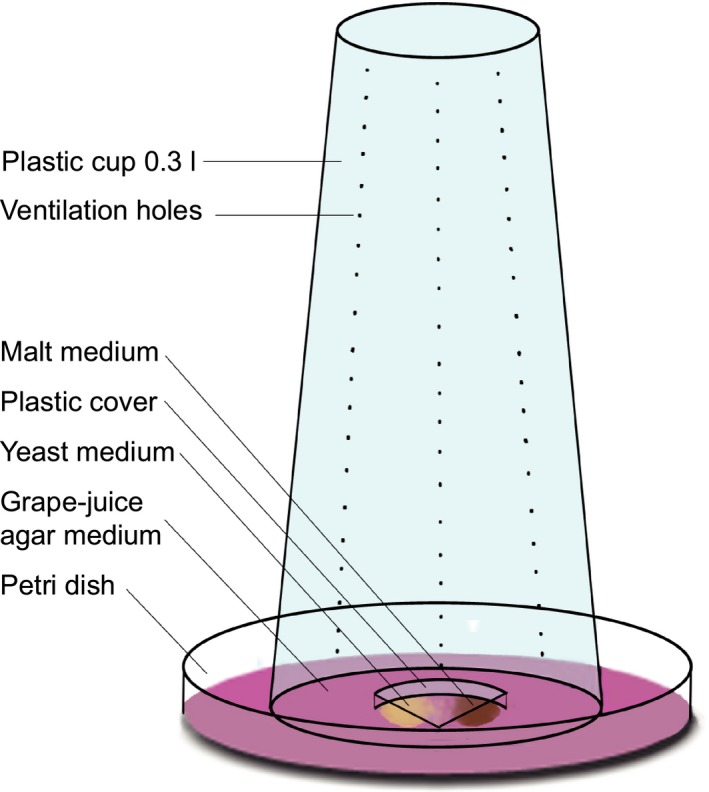
Schematic illustration of a culturing cup. Petri dish with grape‐juice agar medium, dried yeast, and malt medium to enhance oviposition, topped with a perforated plastic cup. Yeast and malt media were covered with a plastic roof that prevented flies from falling into the media from above but allowed access to the media from the side

To allow enough space for mating, batches of up to 100 emerged adults were transferred to perforated transparent 300‐ml plastic cups on 90‐mm Petri dishes (Figure 2) containing 40 ml grape‐juice agar (24% grape juice, 2.5% sucrose, 2% agar, and 0.2% methyl‐4‐hydroxybenzoate in deionized water; modified from Sullivan, Ashburner, & Hawley, 2000). Approximately 0.10 g of dried yeast and 0.30 g of medium (malt for *D. nigrosparsa*, cornmeal for the other species) were added.

### 2.2 Laboratory experiments

N_2013_ was assayed between August 2013 and March 2014. The fifth generation of N_2013_ was used for all assays except for the longevity and oviposition rate assay, where the sixth generation was used. H_2015_, M_2015_, N_2015_, and O_2015_ were assayed between August 2015 and February 2016. All species and replicates were randomly positioned in all experimental setups. For sexing, flies were CO_2‐_anesthetized lightly 48 h before an experiment (Colinet & Renault, 2012) except for the longevity and oviposition rate assays, for which flies were anesthetized right before the experiment. With the exception of thermal tolerance experiments, all 2013 experiments were performed under fluctuating temperatures, and all 2015 experiments were performed at 19°C.

### 2.3 Oviposition rate and longevity

For N_2013_, 50 females and 50 males (4–8 days old) were transferred to culturing cups with grape‐juice agar. New agar plates were provided twice a week after 72 or 96 hr, when also the oviposition rate, the number and sex of dead flies, and the dead flies’ age were determined. Four replicates were assayed for each laboratory population N_2013_K and N_2013_P.

For H_2015_, M_2015_, N_2015_, and O_2015_, four replicates per species were assayed. In each replicate, 30 females and 30 males of similar age (H_2015_ 1–4 days for replicates 1 and 2, and 1–7 days for replicates 3 and 4, M_2015_ 1–3 days, N_2015_ 1–4 for replicates 1–3 and 1–7 days for replicate 4, and O_2015_ 1–4 days) were transferred to each cup. New agar plates were provided four times per week. The number of eggs per female per 24 hr (oviposition rate) was recorded twice a week. The number and sex of dead flies and the dead flies’ age were recorded four times per week until all flies were dead.

### 2.4 Productivity and development time

For N_2013_, five females and five males per line of the same age were allowed to oviposit for 48 hr on malt medium with additional 0.10 g dry yeast as a protein source. The number, sex, and development time of emerged flies were recorded once a week, and the mean number of emerged flies per parental female was calculated. The assay was replicated 11 times for each line.

### 2.5 Larval competition

First‐instar larvae of *D. nigrosparsa* (N_2013_) and *D. subobscura* (ratio 1:2) were transferred into 36‐ml vials containing 5 ml plain agar (2% agar and 0.2% methyl‐4‐hydroxybenzoate in deionized water) at the bottom to provide moisture covered with 3 ml malt medium. *Drosophila subobscura* was used as a competition standard for *D. nigrosparsa* due to its co‐occurrence at the timber line sharing mushrooms as oviposition substrate (Bächli, 1977; Kinzner et al., 2016). Three different densities of *D. nigrosparsa : D. subobscura* (12:24, 24:48, 48:96) were replicated five times per line. Larva‐to‐adult viability, sex, and development time of emerged flies were recorded three times a week, and adults were removed to avoid additional oviposition.

### 2.6 Starvation resistance

For N_2013_, 35 to 38 flies per line and for H_2015_, M_2015_, N_2015_, and O_2015_, 20 females and 20 males of the same age were placed individually in 36‐ml vials that contained 8 ml plain agar to prevent desiccation. The number and sex of dead flies were recorded every 8 hr (06.00, 14.00, 22.00) until all flies were dead. Starvation resistance depicts survival time without access to food.

### 2.7 Heat knockdown

Four to six females of line N_2013_ and four females of H_2015_, M_2015_, N_2015_, and O_2015_ were placed in empty, flat‐bottom 5‐ml glass vials without anesthesia. Foam rubber moistened with 30 μl distilled water was inserted in the vials’ lids. To assess heat resistance along a temperature gradient, vials were transferred into a custom‐built preheated water bath. Water temperature was gradually increased from 25°C to 40°C at 0.5°C/min. Temperature was measured with an electronic thermometer (TFX 430, ebro Electronic GmbH; Ingolstadt, Germany) with an accuracy of 0.05°C. Motionless flies on the bottom of the vial not reacting to tipping were considered knocked down. The number of flies in coma was recorded every 30 s. Forty replicates were tested for each N_2013_ line and for H_2015_, M_2015_, N_2015_, and O_2015_.

### 2.8 Acute critical maximum and minimum temperature

Procedures were similar to those of Overgaard, Kristensen, Mitchell, & Hoffmann (2011). Briefly, for acute critical maximum temperature, flies of similar age (max. 11 days old) were sexed and put on malt medium (N_2013_, N_2015_) or corn medium (M_2015_, O_2015_, and H_2015_). Five *D. nigrosparsa* females per line of N_2013_ and four females per species of H_2015_, M_2015_, N_2015_, and O_2015_ were transferred into an empty 5‐ml vial each without anesthesia. Vials were placed in a water bath at different, constant temperatures (36.0–38.5°C for N_2013_, and 37.0–40.0°C for H_2015_, M_2015_, N_2015_, and O_2015_ with 0.5°C steps) for 5 min each. For each temperature assayed, naïve flies were used and the percentage of flies in coma (defined as for heat knock down) was recorded.

For acute critical minimum temperature, the same protocol as described above was applied; flies were at most 11 (N_2013_) or 13 days (H_2015_, M_2015_, N_2015_, O_2015_) old. Water temperatures ranged from 3.0 to 1.5°C for N_2013_ and from 9.0 to 1.0°C for H_2015_, M_2015_, N_2015_, and O_2015_ with 0.5°C steps.

### 2.9 Statistical analyses

The experiments longevity, productivity, development time, larval competition, starvation resistance, and heat knockdown were analyzed using analysis of variance (ANOVA). The experiments oviposition rate over lifespan and acute critical maximum and minimum temperature were analyzed using analysis of covariance (ANCOVA). The acute critical maximum and minimum temperatures at which 50% of the flies were knocked down (CT_max_ and CT_min_, respectively) were calculated using linear regressions. All analyses were performed in R version 3.1.1 (R Core Team, 2014).

To gain information on the reproducibility of the oviposition rate, the concordance correlation coefficient (CCC) was calculated describing the egg counter's measurement error (Castañeda, Calabria, Betancourt, Rezende, & Santos, 2012). This coefficient is meaningful, easily measured, and similar to intra‐class correlation coefficient, which evaluates a researcher's accurateness and precision. Due to overly high oviposition rates of H_2015_, surpassing 2,000 eggs per replicate per 24 hr, eggs on corn media were analyzed via manual image analysis using Fiji ImageJ 2.0. (Schindelin et al., 2012). Also, for accurate visual counting, a counting grid was placed on the H_2015_ and M_2015_ media after oviposition.

## 3 RESULTS

### 3.1 Oviposition rate

**Figure 3:**
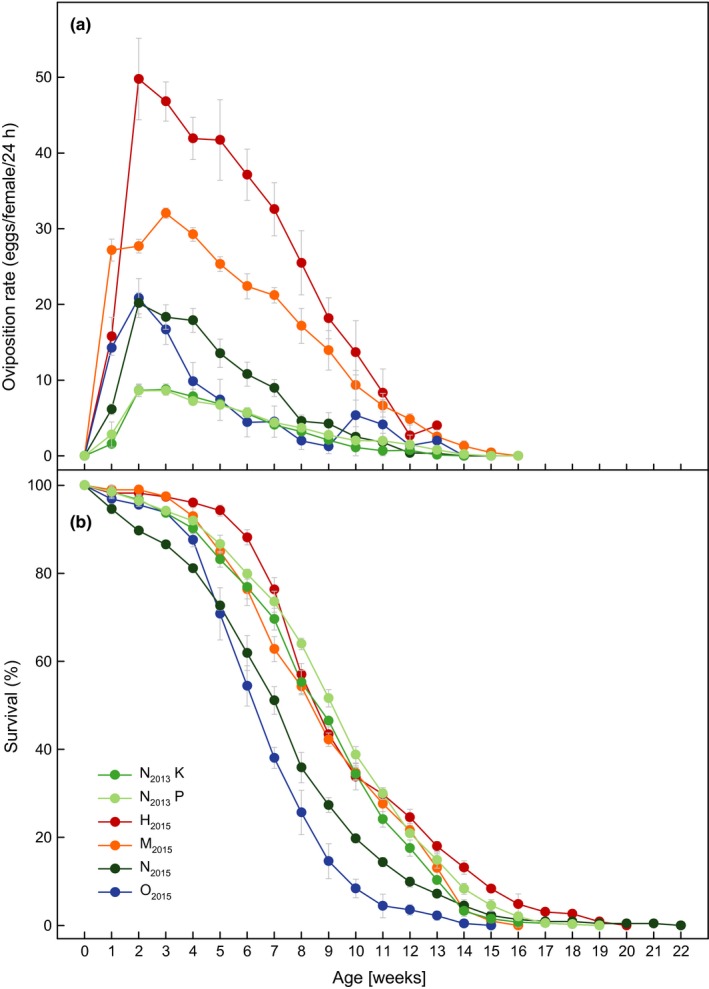
Oviposition rate over life and longevity of four *Drosophila* species. (a) Oviposition rate, weekly mean eggs per female per 24 hr ± standard error. (b) Longevity, weekly mean percentage of surviving flies ± standard error. N_2013_K and N_2013_P flies were kept in a fluctuating temperature regime and assayed in 2013, and all others were kept in a constant temperature regime and assayed in 2015. Abbreviations: N_2013_K, *Drosophila nigrosparsa* Kaserstattalm population (bright green); N_2013_P, *D. nigrosparsa* Pfitscherjoch population (pale green); H_2015_, *Drosophila hydei* (red); M_2015_, *Drosophila melanogaster* (orange); N_2015_, *D. nigrosparsa* (dark green); O_2015_, *Drosophila obscura* (blue)

Counts were accurate with a mean error below 0.4% (CCC analysis, Table S2). Mean oviposition rate over lifespan (averaged per week) was lowest for N_2013_K and N_2013_P (Figure 3a, Data S1), without a significant difference between the two populations (Table 1). Mean oviposition rate was highest for H_2015_ with more than seven times higher rates than for the N_2013_ populations. Mean oviposition rate differed significantly among species at the constant temperature regime (Table 1). Oviposition rate increased steeply at an early age for all species (Figure 3a). N_2013_K, N_2013_P, and M_2015_ reached their maximum in Week 3, whereas H_2015_, N_2015_, and O_2015_ reached their maximum in Week 2 before decreasing gradually.

**Table 1:** Results of the analysis of variance for life‐history and physiological‐limit assays

Assay	Variables	*df*	Sum Sq	Mean Sq	*F*‐value	*p*‐Value
Oviposition rate	N_2013_ population (ANCOVA)	1	1.60	1.60	0.48	.496
	species_2015_ (ANCOVA)	3	2,883.54	961.18	21.33	<.001
Longevity	N_2013_ population within sex	2	3,032.00	1,516.00	2.67	.070
	N_2013_ sex within population	2	20,919.00	10,460.00	18.45	<.001
	species_2015_	3	56,956.90	18,985.60	34.18	<.001
	H_2015_ sex	1	27,424.40	27,424.40	50.86	<.001
	M_2015_ sex	1	5,120.53	5,120.53	9.74	.002
	N_2015_ sex	1	3,955.40	3,955.40	5.925	.016
	O_2015_ sex	1	3,550.12	3,550.12	11.14	.001
Productivity	N_2013_ population within sex	2	3.00	1.52	1.19	.304
	N_2013_ sex within population	2	20.00	10.01	7.88	<.001
Development time	N_2013_ population within sex	2	1,666.00	832.90	7.14	<.001
	N_2013_ sex within population	2	78.00	38.80	0.33	.717
Larval competition–Larva‐to‐adult viability	N_2013_ population within sex within density	6	98.00	16.40	0.39	.887
	N_2013_ sex within population within density	6	91.00	15.10	0.36	.905
Larval competition–Development time	N_2013_ population within sex within density	6	631.00	105.00	3.27	.004
	N_2013_ sex within population within density	6	327.00	54.00	1.69	.121
Starvation resistance	N_2013_ population within sex	2	481.00	241.00	0.23	.797
	N_2013_ sex within population	2	3,418.00	1,708.90	1.61	.201
	species_2015_	3	48,812.60	16,270.90	15.00	<.001
	H_2015_ sex	1	2,037.89	2,037.89	3.97	.054
	M_2015_ sex	1	113.33	113.33	0.04	.845
	N_2015_ sex	1	0.52	0.52	0.01	.976
	O_2015_ sex	1	2,822.40	2,822.40	8.55	.006
Heat knockdown	N_2013_ population	1	0.07	0.07	0.08	.782
	species_2015_	3	129.98	43.33	115.60	<.001
Acute critical maximum temperature	N_2013_ population (ANCOVA)	1	0.01	0.01	0.02	.891
	species_2015_ (ANCOVA)	3	55,729.20	18,576.40	24.48	<.001
	H_2015_–M_2015_ (ANCOVA)	1	656.00	656.00	0.73	.396
	H_2015_–N_2015_ (ANCOVA)	1	32,544.60	32,544.60	37.68	<.001
	H_2015_–O_2015_ (ANCOVA)	1	31,350.40	31,350.40	42.98	<.001
	M_2015_–N_2015_ (ANCOVA)	1	23,959.60	23,959.60	29.87	<.001
	M_2015_–O_2015_ (ANCOVA)	1	22,936.50	22,936.50	34.43	<.001
	N_2015_–O_2015_ (ANCOVA)	1	11.16	11.16	0.02	.895
Acute critical minimum temperature	N_2013_ population (ANCOVA)	1	0.01	0.01	0.07	.796
	species_2015_ (ANCOVA)	3	18,9853.00	63,284.50	105.90	<.001
	H_2015_–M_2015_ (ANCOVA)	1	2,770.09	2,770.09	4.63	.033
	H_2015_–N_2015_ (ANCOVA)	1	144,893.00	144,893.00	260.20	<.001
	H_2015_–O_2015_ (ANCOVA)	1	63328.00	63328.00	106.50	<.001
	M_2015_–N_2015_ (ANCOVA)	1	108,834.00	108,834.00	180.10	<.001
	M_2015_–O_2015_ (ANCOVA)	1	39,825.90	39,825.90	66.10	<.001
	N_2015_–O_2015_ (ANCOVA)	1	13,591.30	13,591.30	24.48	<.001

Analysis of covariance (ANCOVA) was performed where noted in parentheses. Each line represents a single analysis. For oviposition rate, ramping heat knockdown, and acute critical maximum and minimum temperature, only female flies were used, and thus, no sex‐dependent analysis was conducted. N_2013_K and N_2013_P flies were kept in a fluctuating temperature regime and assayed in 2013, and all others were kept in a constant temperature regime and assayed in 2015. Abbreviations: *df*, degrees of freedom; Sum Sq, sum of squares; Mean Sq, mean sum of squares; N_2013_K, *Drosophila nigrosparsa* Kaserstattalm population; N_2013_P, *D. nigrosparsa* Pfitscherjoch population; H_2015_, *Drosophila hydei*; M_2015_, *Drosophila melanogaster*; N_2015_, *D. nigrosparsa*; O_2015_, *Drosophila obscura*.

### 3.2 Longevity

**Figure 4:**
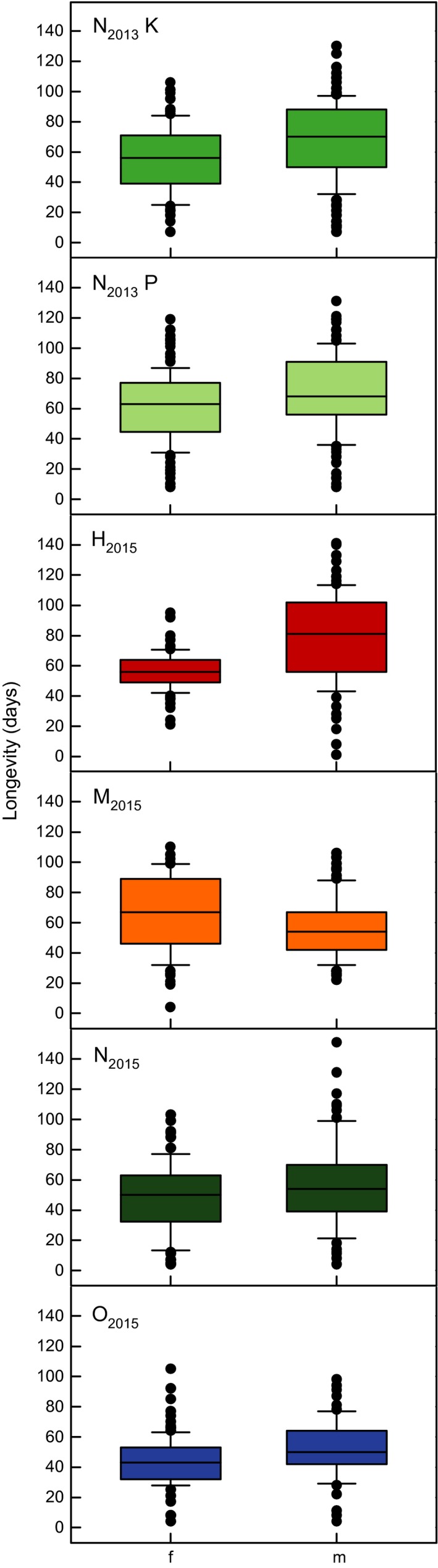
Longevity of four *Drosophila* species for both sexes. N_2013_K and N_2013_P flies were kept in a fluctuating temperature regime and assayed in 2013, and all others were kept in a constant temperature regime and assayed in 2015. Abbreviations: f, females; m, males; N_2013_K, *Drosophila nigrosparsa* Kaserstattalm population (bright green); N_2013_P, *D. nigrosparsa* Pfitscherjoch population (pale green); H_2015_, *Drosophila hydei* (red); M_2015_, *Drosophila melanogaster* (orange); N_2015_, *D. nigrosparsa* (dark green); O_2015_, *Drosophila obscura* (blue)

Mean longevity was highest for H_2015_ males with 78.64 ± 2.83 days survival and lowest for O_2015_ males with 41.94 ± 1.96 days survival (Figure 4, Data S1). Although N_2015_ flies on average lived not extremely long, a few individuals lived longer than 140 days. N_2013_ flies from the fluctuating regime lived longer than N_2015_ flies from the constant regime. Longevity differed significantly between the two N_2013_ populations and among the species of the constant temperature regime (Table 1). Survival rates were similar for all species (Figure 3b): 50% of flies died between Week 6 and 7 (O_2015_), Week 7 and 8 (N_2015_), Week 8 and 9 (N_2013_K, H_2015_, M_2015_), and Week 9 and 10 (N_2013_P). Males of all species lived significantly longer than females, except for M_2015_, of which females lived longer (Figure 4, Table 1).

### 3.3 Productivity and development time

**Figure 5:**
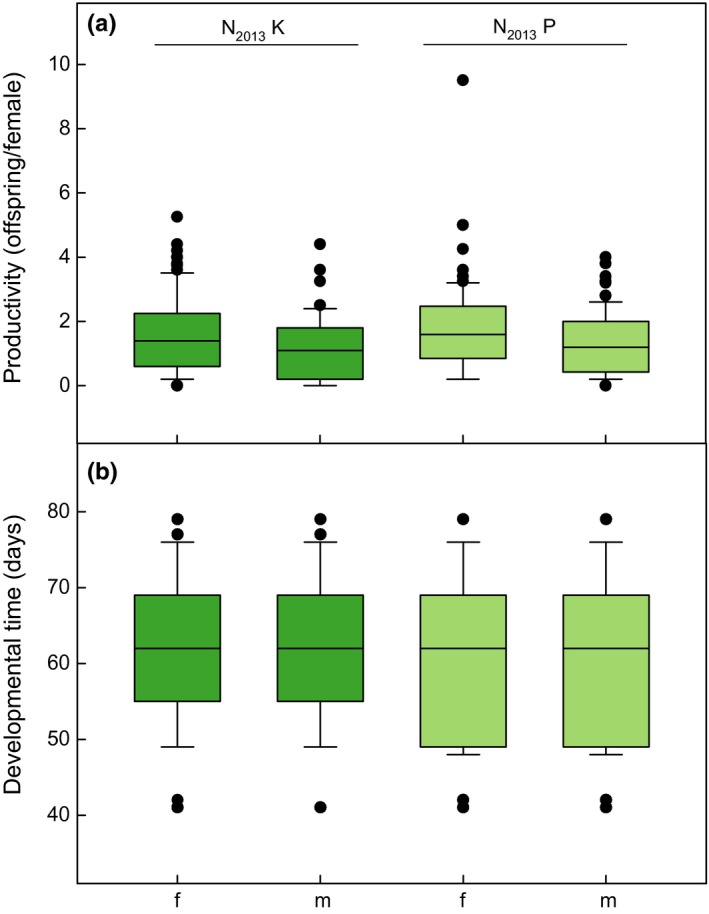
Productivity and development time of *Drosophila nigrosparsa*. (a) Number of female and male offspring per parental female for both N_2013_ populations. (b) Development time of females and males for both N_2013_ populations. N_2013_K and N_2013_P flies were kept in a fluctuating temperature regime and assayed in 2013. Abbreviations: f, females; m, males; N_2013_K, *D. nigrosparsa* Kaserstattalm population (bright green); N_2013_P, *D. nigrosparsa* Pfitscherjoch population (pale green)

For N_2013_K, 1.63 ± 0.13 females and 1.13 ± 0.10 males emerged per parental female (Figure 5a, Data S1). For N_2013_P, 1.79 ± 0.15 females and 1.34 ± 0.88 males emerged per parental female. Productivity did not differ significantly between the two populations (Table 1). Sex ratio of emerged adults (males : females) was 0.68 (N_2013_K) and 0.77 (N_2013_P), that is, significantly more females than males emerged for both populations and within the populations (Table 1). Maximum productivity across all replicates was 5.25 females and 4.40 males for N_2013_K, and 9.50 females and 4.00 males for N_2013_P (Figure 5a). Development time was 62.03 ± 0.41 days (Figure 5b) for N_2013_K females and 61.50 ± 0.48 days for males. It was significantly shorter for N_2013_P (Table 1), with females taking 60.14 ± 0.42 and males 60.18 ± 0.47 days.

### 3.4 Larval competition

**Figure 6:**
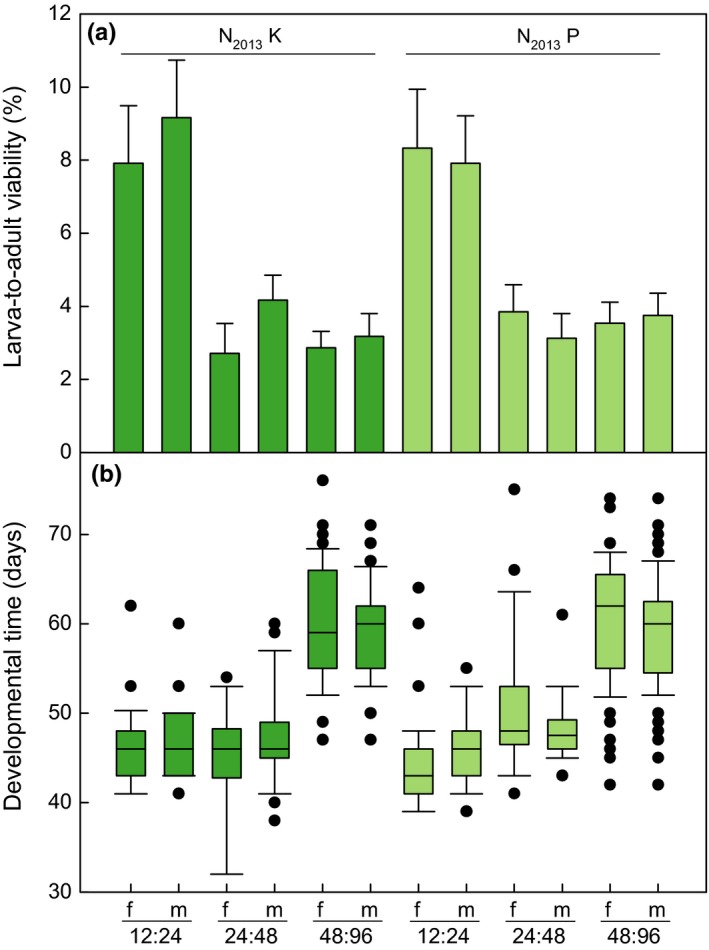
Larval competition between *Drosophila nigrosparsa* and *Drosophila subobscura*. (a) Larva‐to‐adult viability of *D*. *nigrosparsa*, mean percentage of adults eclosed ± standard error. (b) Development time in a competitive situation of females and males for both N_2013_ populations. N_2013_K and N_2013_P flies were kept in a fluctuating temperature regime and assayed in 2013. Abbreviations: f, females; m, males; N_2013_K, *D. nigrosparsa* Kaserstattalm population (bright green); N_2013_P, *D. nigrosparsa* Pfitscherjoch population (pale green)

At density 12:24 N_2013_ *: D. subobscura*, larva‐to‐adult viability for N_2013_ females was about 8% and for males between 8% and 9% (Figure 6a, Data S1). At density 24:48, larva‐to‐adult viability decreased for females to 2%–4% and for males to 3%–4% which remained stable at density 48:96. Density 12:24 differed significantly from the two others, but densities 24:48 and 48:96 as well as the populations and sexes did not differ significantly (Table 1).

At density 12:24*,* development time was about 45 days for both sexes (Figure 6b). At density 24:48, development time increased for population P to 48–50 days, and at density 48:96, development time increased for both populations to about 60 days. For N_2013_K, density 12:24 did not differ significantly from 24:48, but both densities differed significantly from 48:96 (Table 1). For N_2013_P, all densities differed significantly from each other. Populations were significantly different, but sexes were not.

### 3.5 Starvation resistance

**Figure 7:**
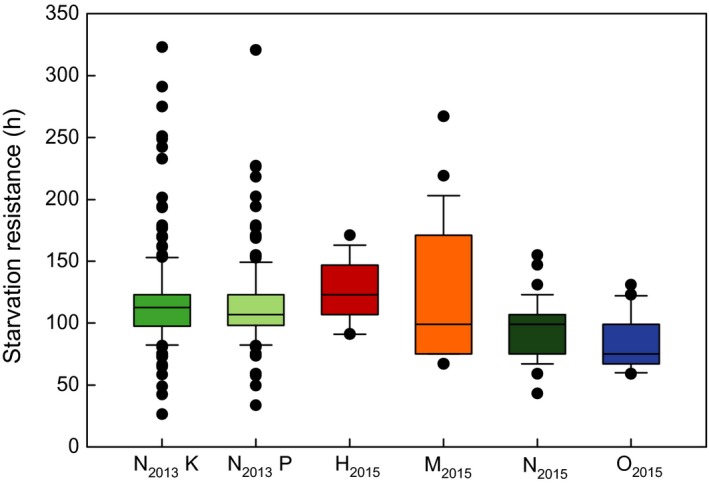
Starvation resistance of four *Drosophila* species. Survival time in hours without access to food. N_2013_K and N_2013_P flies were kept in a fluctuating temperature regime and assayed in 2013, and all others were kept in a constant temperature regime and assayed in 2015. Abbreviations: N_2013_K, *Drosophila nigrosparsa* Kaserstattalm population (bright green); N_2013_P, *D. nigrosparsa* Pfitscherjoch population (pale green); H_2015_, *Drosophila hydei* (red); M_2015_, *Drosophila melanogaster* (orange); N_2015_, *D. nigrosparsa* (dark green); O_2015_, *Drosophila obscura* (blue)

Mean starvation resistance was highest for H_2015_ (125.95 ± 3.82 hr, Figure 7, Data S1) and lowest for O_2015_ (83.00 ± 3.14 hr). Mean starvation resistance differed significantly among the species of the constant regime but not between the populations N_2013_K and N_2013_P nor between the sexes, except for O_2015_ where females lived significantly longer than males (Table 1). N_2013_ reached the most extreme starvation resistance values with maxima of more than 320 hr and minima of about 30 hr (Figure 7).

### 3.6 Heat knockdown

**Figure 8:**
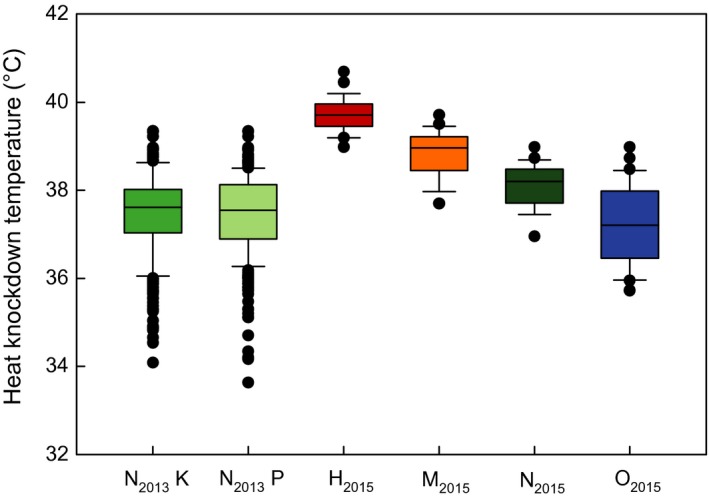
Heat knockdown temperature of four *Drosophila* species. Heat tolerance assayed in a ramping approach. N_2013_K and N_2013_P flies were kept in a fluctuating temperature regime and assayed in 2013, and all others were kept in a constant temperature regime and assayed in 2015. Abbreviations: N_2013_K, *Drosophila nigrosparsa* Kaserstattalm population (bright green); N_2013_P, *D. nigrosparsa* Pfitscherjoch population (pale green); H_2015_, *Drosophila hydei* (red); M_2015_, *Drosophila melanogaster* (orange); N_2015_, *D. nigrosparsa* (dark green); O_2015_, *Drosophila obscura* (blue)

Ramping heat knockdown temperatures (KD_max_) for N_2013_ were 37.46 ± 0.05°C (N_2013_K, Figure 8, Data S1) and 37.44 ± 0.05°C (N_2013_P). KD_max_ was highest for H_2015_ (39.74 ± 0.07°C), followed by M_2015_ (38.80 ± 0.09°C) and N_2015_ (38.11 ± 0.07°C), and was lowest for O_2015_ (37.28 ± 0.15°C). Ramping heat knockdown temperatures differed significantly among the species of the constant regime but not between the populations within N_2013_ (Table 1).

### 3.7 Acute critical maximum temperature

Linear regression revealed a significant relation between increasing temperature and acute heat knockdown for all species and populations (Table 2). At the lowest temperature (36.50°C for the variable regime and 37.00°C for the constant regime), no fly was in coma (Table 2). Fifty percent knock‐down temperature (CT_max_) was at 37.55°C (N_2013_K and N_2013_P), 39.32°C (H_2015_), 39.02°C (M_2015_), 37.68°C (N_2015_), and 37.73°C (O_2015_). H_2015_ and M_2015_ both differed significantly from N_2015_ and O_2015_ (Table 1).

**Table 2:** Linear regression models for acute critical maximum and minimum temperature experiments

Assay	Flies	Slope	Intercept	*R* ^2^	*p*‐Value	CT_max/min_ (°C)
Acute critical maximum temperature	N_2013_K	0.52	−19.03	.81	<.001	37.55
	N_2013_P	0.55	−19.97	.89	<.001	37.55
	H_2015_	0.28	−10.56	.47	<.001	39.32
	M_2015_	0.31	−11.75	.56	<.001	39.02
	N_2015_	0.30	−10.94	.56	<.001	37.68
	O_2015_	0.31	−11.29	.67	<.001	37.73
Acute critical minimum temperature	N_2013_K	−0.41	1.60	.50	<.001	2.70
	N_2013_P	−0.45	1.71	.61	<.001	2.69
	H_2015_	−0.07	1.23	.36	<.001	9.99
	M_2015_	−0.11	1.34	.51	<.001	7.47
	N_2015_	−0.11	0.82	.54	<.001	2.83
	O_2015_	−0.15	1.21	.74	<.001	4.63

CT_max/min_, 50% heat (max) and cold (min) knockdown temperature calculate using the linear models. N_2013_K and N_2013_P flies were kept in a fluctuating temperature regime and assayed in 2013, and all others were kept in a constant temperature regime and assayed in 2015. Abbreviations: N_2013_K, *Drosophila nigrosparsa* Kaserstattalm population; N_2013_P, *D. nigrosparsa* Pfitscherjoch population; H_2015_, *Drosophila hydei*; M_2015_, *Drosophila melanogaster*; N_2015_, *D. nigrosparsa*; O_2015_, *Drosophila obscura*.

### 3.8 Acute critical minimum temperature

Linear regression revealed a significant relation between decreasing temperature and acute cold knockdown for all species and populations (Table 2). At the highest temperature (9.00 C), no fly was in coma. Fifty percent knock‐down temperature (CT_min_) was at 2.70°C (N_2013_K), 2.69°C (N_2013_P), 9.99°C (H_2015_), 7.47°C (M_2015_), 2.83°C (N_2015_), and 4.63°C (O_2015_). The species differed significantly in their acute critical minimum temperature (Table 1).

## 4 DISCUSSION

Life‐history traits are among the most basic and essential characteristics describing a species (Pease & Bull, 1988) and contribute to a broader understanding of an organism. Under climate change, it is necessary to understand a species’ ecology, its possible responses to resulting selection pressures, and its potential for rapid adaptation to environmental alterations (Angilletta & Dunham, 2003; Hoffmann & Sgrò, 2011). Life‐history traits include those involved in responding to extreme conditions, such as heat and cold tolerance and starvation resistance, as well as patterns of reproduction and longevity that can all provide insights into evolutionary adaptations and responses to future conditions (Hoffmann & Sgrò, 2011; Huey et al., 2012).

Successful reproduction is the most important function in an organism's life cycle (Lagadic, Caquet, & Ramade, 1994). The pattern of oviposition rate over life in the species studied here (Figure 3) is similar to that found in other *Drosophila* species (e.g., R'Kha, Moreteau, & David, 1997; Boulétreau‐Merle, Allemand, Cohet, & David, 1982). The mean oviposition rates of the two N_2013_ populations were lower than those of all other flies assessed here, including N_2015_ (Figure 3). This intraspecific variation in *D. nigrosparsa* might reflect different rearing regimes, as rearing temperature is known to strongly influence egg‐laying (Ashburner, Golic, & Hawley, 2005). In our study, the constant temperature regime of 19°C (N_2015_) enhanced oviposition compared with a more natural, fluctuating regime (N_2013_). Nevertheless, in both tests, the number of eggs laid by *D*. *nigrosparsa* was relatively low compared with the other assayed species, except *D. obscura*. The low oviposition rate might be explained by cold adaptation (Carbonell et al., 2017; Kubrak, Nylin, Flatt, Nässel, & Leimar, 2017; Schnebel & Grossfield, 1986). *Drosophila nigrosparsa* has a reproductive diapause depending on photopheriodism similar to *D. littoralis* (Lankinen, 1985). However, there was no indication of diapauses under the thermal and photoperiodic regime used in this study—we cultivated *D. nigrosparsa* for 8 years and never observed a diapause. Unfortunately, there is no information available about the oviposition rate of *D. nigrosparsa* in nature.

Longevity can contribute to the reproductive success of an organism, although senescence is also apparent in *Drosophila* and reduces overall productivity (Figure 3) (Nuzhdin, Pasyukova, Dilda, Zeng, & Mackay, 1997). Generally, males lived longer than females in our study, which might reflect female energy investment in egg production and mating behavior (Chapman, Liddle, Kalb, Wolfner, & Partridge, 1995; Fowler & Partridge, 1989). Similarly, *D. buzzatii* males lived longer in another study (Scannapieco, Sambucetti, & Norry, 2009). However, this is not a universal pattern given that M_2015_ females outlived the males. Differences in experimental conditions can shorten or extend the lifespan of an organism (Aigaki & Ohba, 1984; Helfand & Rogina, 2003), which no doubt accounts for the difference between the N_2013_ and N_2015_ strains of *D. nigrosparsa* (Figure 3). This difference probably reflects the influence of the temperature regime: A constant but warm temperature results in a continuous and relatively high metabolic rate, whereas this is not the case with a fluctuating temperature regime. The latter is more appropriate for alpine species such as *D. nigrosparsa*, as they experience strong daily temperature fluctuations in nature. For *D. montana*, another cold‐adapted species, males lived over 130 days on average (Hoikkala, Saarikettu, Kotiaho, & Liimatainen, 2008), but this assay was at 4°C and therefore not comparable with our study. In any case, species differences were discovered, but no species came close to the known maximum lifespan of some Hawaiian drosophilids which can exceed 9 months (Carson, Hardy, Spieth, & Stone, 1970).

Besides the low oviposition rate, *D. nigrosparsa* (N_2013_) also had a low productivity (Figure 5) when compared with *D. melanogaster* (Ochando & Ayala, 1999) and *D. pseudoobscura* (Gowaty, Kim, Rawlings, & Anderson, 2010). The specific numbers of eclosed flies strongly depend on the study design (Barker, 1973). Furthermore, rearing temperature and mating frequency influence productivity (Barker, 1973; Gowaty et al., 2010). Reproductive output might be negatively correlated with cold resistance (Jenkins & Hoffmann, 1999) and, as *D. nigrosparsa* is cold adapted (Table 1), this may contribute to the low productivity of the N_2013_ populations. Finally, it is imaginable that the culture medium we used is suboptimal for *D. nigrosparsa,* possibly leading to its low productivity in the laboratory. However, we have tested a range of media types for *D. nigrosparsa*, and none of these lead to a high productivity.

Short development time is expected to enhance larval survival and reproductive success (Roff, 2000). Moreover, development is expected to be fast in alpine environments due to a short seasonal growth period (Fischer & Fiedler, 2002). In grasshoppers, there is no difference of development time between low‐elevation and high‐elevation populations (Carron, 1996). However, *D. nigrosparsa* displayed long development times compared with other *Drosophila* species. Egg‐to‐adult development time was, on average, 61 days (Figure 6), while it takes *D. montana*, another cold‐adapted species, about 27 days (Salminen, Vesala, & Hoikkala, 2012) and *D. melanogaster* only 8.5 days (Ashburner et al., 2005) to develop under optimum conditions. At 24°C, *D. hydei* develops within 14 days and *D. virilis* within 18 days (Ashburner et al., 2005). Development time is influenced by environmental conditions such as photoperiod (Salminen et al., 2012) and temperature as well as genetic variation (Norry, Bubliy, & Loeschcke, 2001; Zwaan, Bijlsma, & Hoekstra, 1992). *Drosophila birchii* and *D. serrata* have a longer development time with increasing latitude (Griffiths, Schiffer, & Hoffmann, 2005; Sgrò, Blows, & Noor, 2003), and cold adapted strains of *D. subobscura* have a longer development time than warm‐adapted strains (Santos, Brites, & Laayouni, 2006). If cold adaptation is generally linked to longer development times, this might explain the increased development time of *D. nigrosparsa* in relation to other species.

In natural populations, competition might play a role in the abundance of *Drosophila* species (Grimaldi & Jaenike, 1984). Larval competition influences growth rate, development time, and pre‐adult survival (James & Partridge, 1998), and strong larval competitiveness might have positive effects on these traits. Here, larval competitiveness of *D. nigrosparsa* (N_2013_) was assayed using the sympatric and resource‐sharing fly *D. subobscura* (Kinzner et al., 2016). The percentage of eclosed N_2013_ adults decreased by more than half from the first to the second density ratio, but did not decrease further (Figure 5, Table 1). The media used in our assays may have influenced the hatching rate, but as both species use fungal fruiting bodies as rearing substrate in nature (Kinzner et al., 2016), the influence of the artificial media used here may be similar for the two species. In our experiment, we cannot distinguish between the influence of intra‐ and interspecific competition. Thus, the decreasing emergence success might not only reflect the interspecific competitiveness but also the combined effect of intra‐ and interspecific competition. Shorrocks, Rosewell, Edwards, & Atkinson (1984) assumed that, although there may be competition among different drosophilids in nature, they will probably not oust one another even on a shared resource. It seems that *D. nigrosparsa* larvae are not strong competitors because the percentage of emerged adults at the lowest density was lower than without interspecific competition but with higher intraspecific larval density (80 eggs, ca. 20% hatching success, data not shown). Moreover, *D. nigrosparsa* (N_2013_) does not seem to be competitive in response to an early occupation of resources given its extended development time. Concerning the species’ competitive ability, further research is needed not only on larvae but also on adults.

Ephemeral and fragmented food resources, such as mushrooms, are limited patches (Krijger, Peters, & Sevenster, 2001). Thus, and due to shortage or suboptimal quality of food, starvation might often be experienced in *Drosophila* species, and limited access to food might play an important role in high altitudes (Goenaga, Fanara, & Hasson, 2013). However, in concordance with former studies assuming high tolerance for tropical species (van Herrewege & David, 1997; Karan et al., 1998; Parsons, 1983; Sisodia & Singh, 2010), the tropical (H_2015_) and cosmopolitan species (M_2015_) had the highest starvation resistance times in our study and outperformed the alpine‐montane *D. nigrosparsa*. Matzkin et al. (2009) compared 16 *Drosophila* species in terms of starvation resistance, including *D. melanogaster* and *D. hydei*. Both species had much lower starvation resistance compared with our results. Reasons could be the maintenance of the flies (24°C with 35% relative humidity versus 19°C with 70% relative humidity in our study), the experimental setup, but also the origin of flies.

Temperature affects life‐history traits, and knowledge of basic temperature performance is essential for understanding a species’ biology (Angilletta, 2009; Chown & Terblanche, 2006). Ramping heat knockdown evaluates the impact of increasing temperature on flies, not measuring the lethal temperature but an ecologically more realistic limit, the heat coma (Hoffmann, Dagher, Hercus, & Berrigan, 1997). Generally, heat knockdown performance also depends on the experimental design (Santos, Castañeda, & Rezende, 2011; Terblanche, Deere, Clusella‐Trullas, Janion, & Chown, 2007). In other studies, the mean heat knockdown temperature for *D. melanogaster* was higher than in our assay (Kellermann et al., 2012; Overgaard, Kristensen, Mitchell, & Hoffmann, 2011; Sgrò et al., 2010), possibly because of our rearing regime. However, the heat resistance of *D. hydei* seemed unaffected by temperature regime (Santos et al., 2011), but see Kellermann et al. (2012). Interestingly, *D. obscura* reached a considerably higher value than observed for this species by Kellermann et al. (2012). The results of this study are more similar to those of a preliminary study in our laboratory (Eberl, 2016). A reason for these divergent results could be the different origins of flies. The strain used in the current study originated from a population at the alpine timber line (same location as N_2013_ and N_2015_), whereas the other populations were from lower altitudes in Denmark (Kellermann et al., 2012) and Germany (Eberl, 2016). Sisodia and Singh (Sisodia & Singh, 2010) proposed that intraspecific variation strongly depends on the populations’ environment. For example, *D. melanogaster* populations from a subtropical habitat were less temperature‐stress resistant than populations from temperate regions (David, Allemand, van Herrewege, & Cohet, 1983; Sisodia & Singh, 2010; Stanley & Parsons, 1981). Beppu, Yoshida, & Kimura (1996) suggested that low heat tolerance is a common trait of high‐altitudinal drosophilids. Shadow temperatures rarely exceed 35°C in the mountainous distribution area of *D. nigrosparsa* (Zentralanstalt für Meteorologie und Geodynamik: Klimadaten von Österreich 1971–2000, available from: http://www.zamg.ac.at/fix/klima/oe71-00/klima2000/klimadaten_oesterreich_1971_frame1.htm). However, the knockdown temperatures for *D. nigrosparsa* (37.46 ± 0.05°C N_2013_K, 37.44 ± 0.05°C N_2013_P, and 38.11 ± 0.07°C N_2015_) were surprisingly high for an alpine‐montane species relative to tropical or widely spread temperate species in our study as well as in others (e.g., Overgaard, Kristensen, Mitchell, & Hoffmann, 2011). Kellermann et al. (2012) showed that the upper thermal limits of drosophilids are linked to the phylogeny rather than to environmental factors at the species’ main distribution range. However, *D. nigrosparsa* was not part of their study, and we have only limited information about this species’ exact position in the phylogeny of Drosophilidae (Cicconardi et al., 2017), as molecular information on many drosophilids is lacking. The variation among individuals was high for N_2013_K and N_2013_P relative to the four other treatments tested (Figure 7). This might be explained by higher genetic variation in the former, which were founded by 100 females and males per population, whereas all others were founded by single pairs of flies. However, we have no genetic data to confirm this assumption. After all, the relatively high heat resistance of *D. nigrosparsa* could be adaptive—it may allow the species to stay in the sun and microhabitats that can heat up beyond 35°C (Franz, 1979), which could be relevant because of, for example, biotic interactions or competition (Gilman, Urban, Tewksbury, Gilchrist, & Holt, 2010).

Physiological adaption to extreme temperatures is an important limiting factor to an ectotherm's distribution (Andersen et al., 2015; Overgaard, Kearney, & Hoffmann, 2014), especially affecting individuals living in extreme habitats like the Alps. The acute critical maximum temperature, where 50% of females fell in coma, was lower for *D. nigrosparsa* (N_2013_ and N_2015_) than for habitat generalist *Drosophila* species such as *D. pseudoananassae* and *D. simulans* (Overgaard, Kristensen, Mitchell, & Hoffmann, 2011). Concerning the generalist species, both *D. hydei* and *D. melanogaster* were more sensitive to heat in this than in another study (Overgaard, Kristensen, Mitchell, & Hoffmann, 2011). Our results of the acute critical maximum temperatures reflect the ramping heat knockdown results (Figure 8). The acute critical minimum temperature for *D. nigrosparsa* was similarly low at the two culturing temperatures (N_2013_, N_2015_; Tables 1 and 2). *Drosophila hydei* and *D. melanogaster* had considerably weaker cold resistance. Overgaard, Kristensen, Mitchell, & Hoffmann, (2011) observed lower values for both species. Other generalist drosophilids, such as *D. pseudoananassae*, were more vulnerable to cold temperatures, and *D. simulans* was close to *D. melanogaster* (Overgaard, Kristensen, Mitchell, & Hoffmann, 2011). *Drosophila obscura*, a temperate species with wide distribution, was more cold resistant than all mentioned species except *D. nigrosparsa*. The environmental conditions at high elevations, such as decreasing temperature with increasing altitude and rapid variations in temperature and distinctive seasonality (Barry, 1992), might enforce better cold resistance, so that alpine species can withstand colder temperatures than tropical or lowland temperate species. Moreover, development under variable thermal conditions might widen the thermal limits (Overgaard, Hoffmann, & Kristensen, 2011).

*Drosophila nigrosparsa* seems well adapted to the harsh conditions at high altitudes as the range between upper and lower thermal limit is larger than that of any other species tested here (N_2013_ 2.70–37.55°C, N_2015_ 2.83–37.68°C), enabling it to greatly withstand variable temperatures in higher or northern regions (Calosi, Bilton, Spicer, Votier, & Atfield, 2010; Goto & Kimura, 1998). The tropical and cosmopolitan species were less robust. The challenging alpine climate has most likely selected for a wide range of temperature tolerance (Gaston & Chown, 1999; Goto, Yoshida, Beppu, & Kimura, 1999). Seasonality also plays a major role in evolution, and cold tolerance is key to selection in temperate or arctic (Goto et al., 1999) and most likely also alpine regions. In conclusion, the alpine *D. nigrosparsa* can withstand relatively high temperatures compared with drosophilids from warmer origins like *D. melanogaster* and *D. hydei*. Climate change research predicts warmer winters and warmer conditions in general (IPCC, 2007). Nevertheless, what extent of temperature rise species can withstand does not necessarily lead to any conclusion about long‐term effects or impacts on productivity or food resources. Moreover, biological interactions strongly influence species’ distributions, which are also changed by climatic alteration (Davis, Jenkinson, Lawton, Shorrocks, & Wood, 1998). Kellermann et al. (2012) suggested that drosophilids cannot easily evolve to increase upper thermal tolerance. Further, the study suggested quite small temperature safety margins (deviation of temperature maxima at place of origin and thermal limit) for tropical species, which thus might be particularly vulnerable to climate change (Kellermann et al., 2012). Temperate species might be least affected (Kellermann et al., 2012). The vulnerability of alpine species is still unknown but alpine environments are thought to be particularly susceptible (IPCC, 2007). Moreover, a high extinction rate for species adapted to cool habitats was observed (Bernardo & Spotila, 2006; Sinervo et al., 2010). Thus, *D. nigrosparsa* and other alpine species might be among the most affected organisms with ongoing climate warming.

Referring to our main question, the life‐history traits and physiological limits of the alpine fly *Drosophila nigrosparsa* seem to be, at least partly, a result of the harsh mountain environment. On the one hand, the low oviposition rate and productivity as well as elongated development time and pronounced cold resistance reflect the cool conditions at high altitudes; a relatively high heat resistance and the resulting large thermal tolerance range reflect extreme temperature variability. On the other hand, we cannot connect longevity, competitiveness, and starvation resistance with the alpine environment. All results are snapshots of the current states of the traits measured—life‐history traits and physiological limits may change in the future through evolution or physiological plasticity as climate change effects become apparent. Future investigations of these traits of natural populations might help drawing a more detailed picture of *D. nigrosparsa* in its alpine environment.

## CONFLICT OF INTEREST

None declared.

## AUTHORS CONTRIBUTION

AAH, JS, WA, BCSS, FMS planned the experiments and created the conception; M‐CK, PK, MN, CH, SE performed the experiments; M‐CK, PK, MN, JS performed the statistical analyses; M‐CK, PK, MN, WA, BCSS, FMS wrote the manuscript. M‐CK, PK, MN, CH, SE, AAH, JS, WA, BCSS, FMS revised and approved the manuscript.
