# Supplementary material for: Life‐history traits and physiological limits of the alpine fly Drosophila nigrosparsa (Diptera: Drosophilidae): A comparative study
Source: Ecol Evol. 2018 Jan 17;8(4):2006–20. doi: 10.1002/ece3.3810 (PMC5817156; doi:10.1002/ece3.3810)
Supplement: Supplementary file 1 [file ECE3-8-2006-s001.doc]

***S1 Table.*** ***Temperature regime used in environmental test chambers for cultivation of Drosophila nigrosparsa.***

| **Daytime [hh:mm]** | **Temperature [°C]** | **Light** |
| --- | --- | --- |
| 01:00 - 05:00 | 7 | no |
| 05:00 - 08:00 | 14 | yes |
| 08:00 - 10:00 | 18 | yes |
| 10:00 - 12:00 | 22 | yes |
| 12:00 - 14:00 | 25 | yes |
| 14:00 - 15:00 | 27 | yes |
| 15:00 - 17:00 | 20 | yes |
| 17:00 - 19:00 | 20 | yes |
| 19:00 - 21:00 | 15 | yes |
| 21:00 - 01:00 | 10 | no |
