# Supplementary material for: Life‐history traits and physiological limits of the alpine fly Drosophila nigrosparsa (Diptera: Drosophilidae): A comparative study
Source: Ecol Evol. 2018 Jan 17;8(4):2006–20. doi: 10.1002/ece3.3810 (PMC5817156; doi:10.1002/ece3.3810)
Supplement: Supplementary file 2 [file ECE3-8-2006-s002.doc]

**S2 Table. Concordance Correlation Coefficient (CCC) of egg counting for the oviposition rate assay.** CCC results broken down on species and media. Flies were kept in a constant temperature regime and assayed in 2015. Abbreviations: H_2015_, *D. hydei*; M_2015_, *D. melanogaster*; N_2015_, *D. nigrosparsa*; O_2015_, *D. obscura*.

| **Species** | **all media** | **agar** | **yeast** | **corn/malt** |
| --- | --- | --- | --- | --- |
| H_2015_ | 0.9981 | 0.9953 | 0.9526 | 0.9960 |
| M_2015_ | 0.9985 | 0.9904 | 0.9958 | 0.9969 |
| N_2015_ | 0.9960 | 0.9973 | 0.9965 | 0.9904 |
| O_2015_ | 0.9941 | 0.9987 | 0.9966 | 0.9919 |
| Species_2015_ | 0.9983 |  |  |  |
